# Supplementary material for: Automatic design of mechanical metamaterial actuators
Source: Nat Commun. 2020 Aug 20;11:4162. doi: 10.1038/s41467-020-17947-2 (PMC7441157; doi:10.1038/s41467-020-17947-2)
Supplement: Supplementary file 1 — Supplementary Information [file 41467_2020_17947_MOESM1_ESM.pdf]

— Supplementary Information —

Automatic Design of Mechanical Metamaterial Actuators

Bonfanti et al.

Supplementary figures

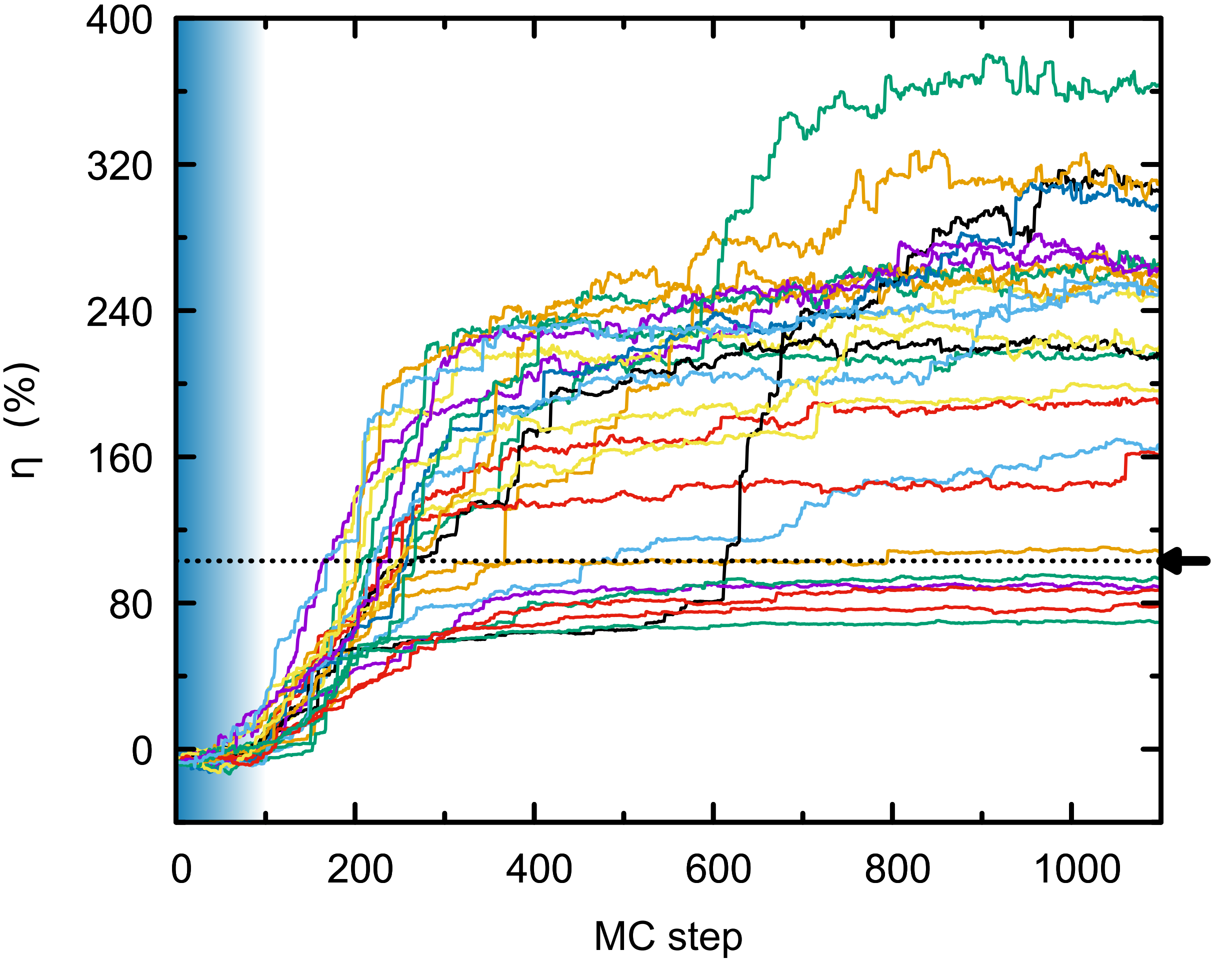

Supplementary Figure 1: **Efficiency evolves across different paths.** Plot of instantaneous η during MC dynamics for the orthogonal motion case. Each curve is obtained with a different initial random seed. Shaded area highlights the annealing phase in the first 100 MC steps (see Method). Only the efficiencies corresponding to accepted MC steps are reported. Arrow and dashed line mark the efficiency of the human-designed structure.

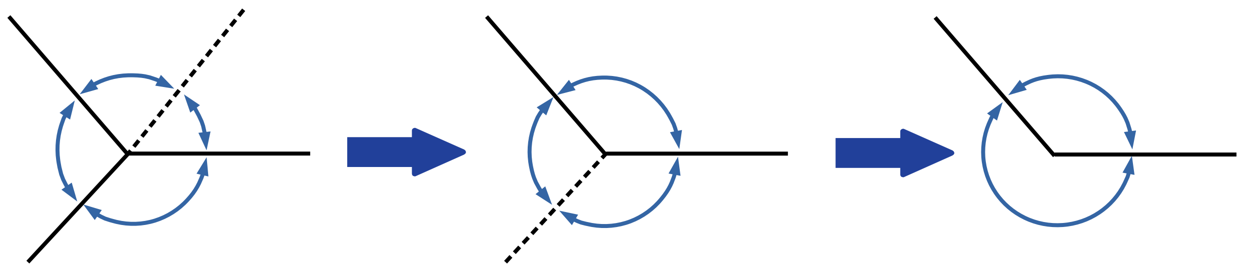

Supplementary Figure 2: **Angular springs re-neighboring.** When a beam is removed (dashed line), the two angular springs connecting to that beam are removed, and a new spring is formed between the newly neighboring beams.

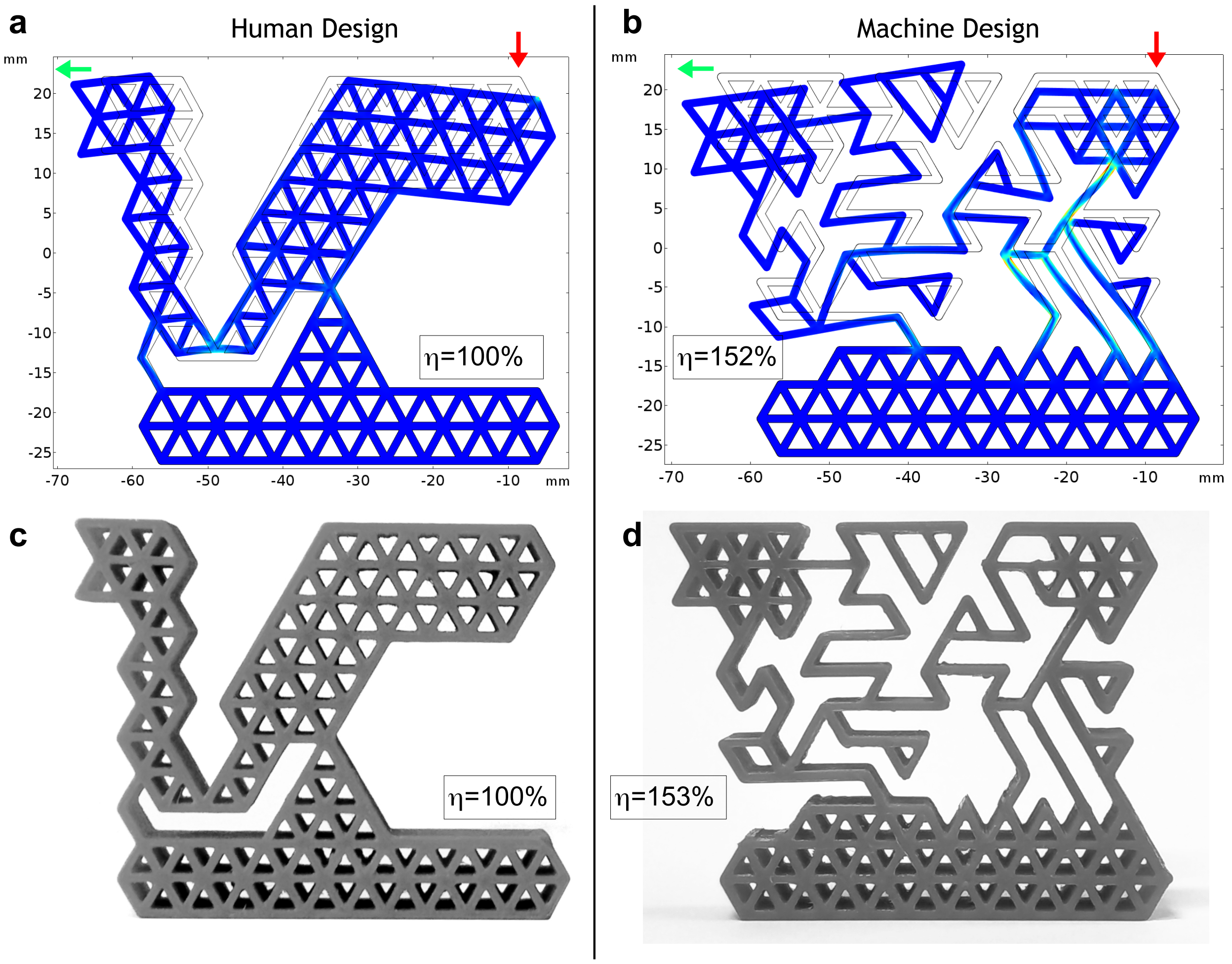

Supplementary Figure 3: **Automatic design achieves efficient orthogonal movement.** Comparison of (a) human-designed and (b) machine-designed structures for FEM simulated and (c,d) the corresponding 3D printed realizations. Color gradient reports the Von Mises stress with the same colorbar as in Fig. 1c. Resulting efficiencies are reported in each panel. For movement visualization see Supplementary Movie 3.
