# Supplementary material for: Automatic design of mechanical metamaterial actuators
Source: Nat Commun. 2020 Aug 20;11:4162. doi: 10.1038/s41467-020-17947-2 (PMC7441157; doi:10.1038/s41467-020-17947-2)
Supplement: Supplementary file 3 — Description of Additional Supplementary Files [file 41467_2020_17947_MOESM3_ESM.pdf]

Description of Additional Supplementary Files

File Name: Supplementary Movie 1
Description: Automatized generation of a metamaterial structure with orthogonal input-output movement. Each frame corresponds to a MC accepted step during the DEM optimization.

File Name: Supplementary Movie 2
Description: Comparison of human and automatic design functioning of 3D printed metamaterial actuators for orthogonal input-output movement.

File Name: Supplementary Movie 3
Description: Comparison of human and automatic design functioning of 3D printed metamaterial actuators for anti-parallel input-output movement.
